# Self-Management Ability Questionnaire Validation in Portuguese Adults With Periodontitis

**DOI:** 10.1016/j.identj.2023.06.003

**Published:** 2023-07-11

**Authors:** Eloïse Gobin, Catarina Izidoro, Patrícia Lyra, Mariana Morgado, Ricardo Castro Alves, José João Mendes, João Botelho, Vanessa Machado

**Affiliations:** a Clinical Research Unit (CRU), Egas Moniz Center for Interdisciplinary Research, Egas Moniz School of Health and Science, Almada, Portugal; b Evidence-Based Hub, Egas Moniz Center for Interdisciplinary Research, Egas Moniz School of Health and Science, Almada, Portugal

**Keywords:** Periodontitis, Periodontal disease, Self-management, Medical management, Patient-reported outcomes

## Abstract

**Objectives:**

We aimed to test the psychometric validity of the adapted and translated Self-Management Ability Questionnaire-Short Form (SMAQ-12) to Portuguese.

**Methods:**

The translation and adaptation of the SMAQ-12 followed international guidelines. We included 280 participants with chronic periodontitis from the Department of Periodontology of the Egas Moniz Dental Clinic. Participants completed the Portuguese version of the SMAQ-12 (SMAQ-12-PT), a 12-item scale with 3 conceptual domains (Role Management, Medical Management, and Emotional Management). The validity of the content, its construct and internal consistency, as well as test–retest reliability were used to estimate psychometric properties.

**Results:**

The SMAQ-12-PT showed an interclass correlation coefficient value of 0.90, with a 95% confidence interval (0.79–0.95; *P* < .001) and high reliability (Cronbach alpha coefficient ranging between 0.78 and 0.94). Confirmatory factor analysis revealed adequate model fit, with comparative fit index of 0.853, goodness-of-fit of 0.947, and a 0.052 value of root mean squared error of approximation.

**Conclusions:**

The SMAQ-12-PT was found to be a valid and reliable instrument in the Portuguese population. Disease management representation for the specialty of periodontology and its impact on periodontal schedules and practices should be evaluated in future studies.

## Introduction

Periodontal maintenance (PM) aims to prevent the progression of periodontitis and tooth loss and is influenced by several factors and conditions.^1^, ^2^, ^3^, ^4^, ^5^, ^6^, ^7^ However, compliance is a key factor in ensuring the effectiveness of PM.^8^, ^9^, ^10^ Patients who are compliant with long-term PM often do not experience tooth loss, with an estimated average loss of 1 tooth per patient every 10 years.^11^

As part of disease treatment compliance, self-management is an individual's ability to manage their disease and included 3 domains: Role Management, Medical Management, and Emotional Management.^12^ Self-Management is considered a critical step in PM^13^ and may improve periodontal health in the long term.^14^^,^^15^. Having this in consideration, Chen et al developed a new psychometric tool to assess periodontitis self-management, the Self-Management Ability Questionnaire (SMAQ).^16^ The original version of the SMAQ consists of 24 items, but its extension raised some concerns about the patient's willingness to complete it, and a shorter version of 12 items was proposed and validated.^13^ This shorter article consists of 3 domains as defined in self-management by Corbin and Strauss^12^: Medical Management (monitoring, treatment compliance, managing information, and selecting appropriate oral cleaning products); Role Management (the ability to change behaviours towards the condition); and Emotional Management (the ability to manage negative emotions and express them in people with chronic periodontitis).^16^

Considering the potential impact of the SMAQ, the need for further validation worldwide, and the discussed need to confirm whether the SMAQ-12 is comprehensive enough to assess the self-management ability of patients with periodontitis,^13^ the validation to other languages is strongly encouraged. In this study, we investigated the psychometric validity of the SMAQ-12 in Portuguese (from Portugal), named SMAQ-12-PT.

## Material and methods

### Design and participants

This study was developed at the Department of Periodontology of the Egas Moniz Dental Clinic (EMDC) with consecutive pool of patients, a Portuguese university dental clinic (Almada, Portugal). The Institutional Review Board approved this study (Ethics Committee of Egas Moniz, ID: 1050) that followed the Declaration of Helsinki of 1975, as revised in 2013.

To be eligible for participation, individuals were required to be 18 years of age or older, Portuguese speakers, diagnosed with periodontitis, and in a specific phase of the periodontal treatment or maintenance. If eligible, each person was invited to participate and signed an informed consent form. The raw data supporting the conclusions of this article are freely available at the following link: https://zenodo.org/record/8010775.

### Cross-cultural adaptation of the SMAQ

The short form of the SMAQ is a 12-item instrument divided into 3 subscales: Role Management (items 1 to 5, ranging from 0 to 20), Medical Management (items 6 to 9, ranging from 0 to 16), and Emotional Management (items 10 to 12, ranging from 0 to 12) (Table 1).^13^ Responses to the items are given on a 5-point Likert scale, ranging as follows: 0 = never; 1 = rarely; 2 = sometimes; 3 = frequently; and 4 = very frequently (Supplementary Table S1). Total scores ranged from 0 to 48 after recording, with higher total scores indicating a greater degree of self-management.

**Table 1:** Test–retest reliability using ICC for the SMAQ-12-PT questionnaire.

	Cronbach *α* coefficient (95% CI)	*P* value*	ICC (95% CI)	*P* value*
**Subscales**				
Medical Management	0.94 (0.87–0.98)	<.001	0.87 (0.71–0.94)	<.001
Role Management	0.91 (0.72–0.98)	<.001	0.84 (0.68–0.92)	<.001
Emotional Management	0.78 (0.32–0.92)	<.001	0.65 (0.36–0.82)	<.001
**Total score**	0.90 (0.77–0.97)	<.001	0.90 (0.79–0.95)	<.001

ICC, intraclass correlation coefficient.

⁎ *P* < .05 mean statistical significance. The *P* values for Cronbach alpha coefficient and ICC indicate that a small *P* value (eg, *P* < .05) indicates a good internal consistency and good agreement between the measurements, for Cronbach and ICC, respectively.

Initially, 2 independent bilingual individuals fluent in both Portuguese and English translated the original English version of the SMAQ-12 into Portuguese (VM and JB) in a “double-blind” process. There was no disagreement, and a final single translated version was produced, named SMAQ-12-PT. Second, the translated version was back-translated to English to confirm the semantic and conceptual equivalence (Supplementary Table S1).

The SMAQ-12-PT test–retest was carried out on a random sample of 28 individuals (10% of the total sample required for validation). The inclusion of this pilot-test sample followed the exact inclusion criteria established previously. In addition to reliability (eligibility criteria are present in the subsection “Design and Participants”), the pilot test was also designed to provide feedback on semantics, which occurred uneventfully. Participants completed the SMAQ-12-PT on 2 occasions, 1 week apart. Validity and reliability assessments were conducted between August and September 2022. The validation phase was carried out between September 2022 and February 2023.

### Variables

Sociodemographic characteristics were collected from a self-report questionnaire. We collected information regarding age, sex, education level (elementary, high school, or higher), marital status (single, married, divorced, and widowed), and employment status (employed, unemployed, or retired).

Amongst the participants habits variable, we included smoking habits (categorised as nonsmokers, former smokers, and active smokers), brushing habits (never, <1 time/d, 1 time/d, 2 times/d, 3 times/d or more), interproximal hygiene in a week (from 0 to 7), last dental visit (<6 months, 6–12 months, >12 months).

Participants’ periodontal information was retrieved from the Periodontology Department of CDEM. All examiners were calibrated. The inter-examiner correlation coefficients, at subject level, ranged from 0.73 to 0.86 and between 0.81 and 0.94, for average periodontal pocket depth (PPD) and clinical attachment loss (CAL), respectively.

Periodontal diagnosis was based on a full-mouth protocol that included clinical parameters circumferentially using a CP-12 manual periodontal probe (Hu-Friedy). PPD, CAL, and bleeding on probing (BOP) were recorded at 6 sites per tooth (mesiobuccal, buccal, distobuccal, mesiolingual, lingual, and distolingual). PPD included the distance from the free gingival margin to the bottom of the pocket and recession (REC) as the distance from the cementoenamel junction (CEJ) to the free gingival margin, and this assessment was given a negative sign if the gingival margin was located coronally to the CEJ. CAL was calculated as the algebraic sum of the REC and PPD measurements for each site. Measurements were rounded to the nearest whole millimeter. Tooth mobility was further assessed according to Lindhe et al.^17^ Furcation involvement (FI) was assessed using a Naber probe^18^ and categorised accordingly.

Periodontitis cases were defined according to the AAP/EFP 2018 consensus, where a patient had periodontitis if interdental CAL was detected in ≥2 mm of nonadjacent teeth or buccal or oral CAL ≥3 mm with pocketing >3 mm was detected in ≥2 teeth.^19^ Each case was then staged and graded each case according to the case definition.^19^

### Sample size calculation

We applied the strategy proposed by Terwee et al^20^ to determine the minimum number of participants required. A total minimum number of 280 participants was needed to allow stability of the variance/covariance matrix when performing confirmatory factor analysis (CFA). This sample did not include participants from the pilot test group of patients.

### Statistical analyses

#### Reliability of SMAQ-12-PT

Using the R version 1.0.8 (R Studio Team 2018), we examined internal consistency through Cronbach alpha (α) coefficient using the “ltm” package. An α coefficient of at least 0.70 was defined as acceptable.^21^ We also assessed the intraclass correlation coefficient (ICC) using the “irr” package and interpreted as follows: excellent (≥0.9), acceptable (0.80–0.89), weak (0.6–0.79), and inexistent (<0.60).^22^

#### Descriptive analysis and construct validity

Results of each subscale and domain are presented as counts and corresponding percentages (%), means and standard deviations (SD), medians and interquartile ranges (IQRs), and minimum and maximum values. The statistical analysis was performed using the R “plyr” package.

CFA was performed using the “lavaan” package to compute the factorial loads and model fit of each subconstruct. Maximum likelihood method was used to compute the model, and differences between models were explored through Chi-square (*χ*^2^) and likelihood ratio test. To test the fitness of CFA, we used the following: *χ*^2^/*df* ratio (good adjustment with values <2),^23^ root mean squared error of approximation (RMSEA; a good model adjustment considered for values between 0.05% and 0.10%; 90% confidence interval [CI]),^24^ confirmatory fit index (CFI; cutoff criterion of ≥0.90 indicates a good fit),^25^ goodness-of-fit (GFI) statistics (values of 0.90 or greater indicate well-fitting models),^26^ and normed-fit index (NFI; cutoff criterion of 0.90).^27^

Then, we analysed the sex invariance of the SMAQ-12-PT. To do this, we estimated 4 consecutive models: (1) unconstrained; (2) factor-loading-constrained (model 1); (3) factor-loading- and structural-covariance-constrained (model 2); and (4) factor-loading-, structural-covariance-, and measurement-residual-constrained (model 3). We were able to obtain the delta values for CFI delta values (ΔCFI) and Chi-square (Δ*χ*^2^). We defined a cutoff point ΔCFI <0.01 as the presence of invariance^25^^,^^28^ and Δ*χ*^2^ <0.095 as invariance between the models.^29^^,^^30^ In addition, we examined the relationships between the SMAQ-12-PT items using Spearman rank correlation coefficient (rho).

The level of statistical significance was set at 5% for all inferential analyses.

## Results

### Reliability

Following a successful inclusion of all estimated participants to test reliability, 17 (60.7%) were female and 11 (39.3%) were male, with similar age intervals (female: 48.9 ± 12.1 vs male: 50.2 ± 5.0, as mean ± SD). The median total score of the SMAQ-PT questionnaire was 31 (range, 24–39).

The overall internal consistency was 0.90 (95% CI, 0.77–0.97), whilst each subscale showed excellent coefficient values for all subscales (Table 1). In addition, ICC analyses showed an overall result of 0.90 (95% CI, 0.79–0.95; *P* < .001), with all subscales reporting values above 0.85, between 0.87 and 1.00. Nominally, one subscale had excellent reliability (Emotional Management subscale = 1.00), and the remaining had acceptable reliability (Table 1 and supplementary file Table S2).

### Characteristics of participants

Overall, this validation phase involved 293 periodontitis cases, with 13 participants being excluded due to missing information. A total of 280 participants completed the SMAQ-PT questionnaire, with a mean age of 60.6 ± 11.8 years, ranging from 28 to 85 years (60.3 ± 10.9 and 60.8 ± 12.6 for females and males, respectively, as mean ± SD). The group was predominantly composed of male participants (51.8%, n = 145; Table 2). Overall, the majority had stage III and IV periodontitis (80.3%), grade B (55.4%) and with generalised extent (57.1%). Most participants were in a diagnosis stage of treatment, 24.3% were active smokers, and 26.4% had never performed interproximal hygiene in the past 7 days.

**Table 2:** Sociodemographic data of included participants (N = 280).

Variable	Result
Age, mean (SD) [min–max], y	60.6 (11.8) [28–85]
Female, % (n)	48.2 (135)
Periodontitis staging, % (No.)	
I	3.6 (10)
II	16.1 (45)
III	33.2 (93)
IV	47.1 (132)
Periodontitis grading, % (No.)	
A	6.4 (18)
B	55.4 (155)
C	38.2 (107)
Periodontitis extent, % (No.)	
Localised	42.9 (120)
Generalised	57.1 (160)
Stage of treatment	
Diagnosis	51.4 (144)
Evaluation	34.3 (96)
Periodontal maintenance	14.3 (40)
Smoking, % (No.)	
Never	39.3 (110)
Former	36.4 (102)
Active	24.3 (68)
Toothbrushing times per day, % (No.)	
0	0.4 (1)
1	8.2 (23)
2	61.4 (172)
3 or more	30.0 (84)
Interproximal hygiene on the last 7 days, % (No.)	
0 times	26.4 (74)
1–7 times	73.6 (206)

The highest mean scores of the SMAQ-PT were observed in items 4 and 6 with 3.1 ± 1.0 and 3.1 ± 0.8, respectively. Items 5 and 11 had the lowest scores with 1.0 ± 1.3 and 1.0 ± 1.1, respectively (Table 3).

**Table 3:** Test–retest reliability using ICC for the SMAQ-12-PT questionnaire.

	Mean (SD)	Median (IQR)	min–max
**SMAQ-12-PT Total Score**	27.5 (6.0)	28 (8)	12–46
Item 1	2.3 (1.4)	3 (2)	0–4
Item 2	3.0 (1.0)	3 (2)	0–4
Item 3	3.0 (1.1)	3 (1)	0–4
Item 4	3.1 (1.0)	3 (1)	0–4
Item 5	1.0 (1.3)	0 (2)	0–4
Item 6	3.1 (0.8)	3 (1)	0–4
Item 7	3.0 (0.9)	3 (2)	1–4
Item 8	2.0 (1.2)	2 (2)	0–4
Item 9	1.5 (1.7)	1 (3)	0–4
Item 10	1.7 (1.3)	2 (3)	0–4
Item 11	1.0 (1.1)	1 (2)	0–4
Item 12	2.8 (1.1)	3 (2)	0–4
***SMAQ-12-PT Domain***			
**Medical Management**	12.3 (3.7)	12 (5)	2–20
**Role Management**	9.6 (2.5)	10 (4)	4–16
**Emotional Management**	5.5 (2.0)	6 (3)	0–12

### SMAQ-12-PT validity

#### Factor validity

The CFA analysis confirmed the structure of the SMAQ (Table 4). The first-order model showed an adequate model fit: GFI = 0.947; CFI = 0.853; RMSEA = 0.052; 90% CI (0.034–0.070) (Table 4).

**Table 4:** Model fit indices in the original model and configurational invariance by sex.

Description	*χ*^2^	*df*	*χ*^2^/*df*	CFI	GFI	RMSEA (90% CI)	ΔCFI	Δ*χ*^2^	Δ*df*
Original model	89.797*	51	1.76	0.853	0.947	0.052 (0.034–0.070)	-	-	-
**Measurement invariance across sex**									
Unconstrained	170.01*	102	1.67	0.769	0.987	0.069 (0.055–0.091)	-	-	-
Model 1	180.16*	111	1.62	0.765	0.986	0.067 (0.054–0.088)	0.030	10.15	9
Model 2	197.98*	120	1.65	0.735	0.985	0.068 (0.051–0.085)	0.030	17.82	9
Model 3	197.98*	120	1.65	0.735	0.985	0.068 (0.051–0.085)	0.000	0.0	0

*χ*^2^, chi-square; *df,* degrees of freedom; CFI, comparative fit index; GFI, goodness-of-fit index; RMSEA, root mean square error of approximation; Δ, delta.

#### Psychometric analysis

The SMAQ-12-PT demonstrated adequate reliability with a Cronbach *α* coefficient of 0.90 (95% CI, 0.77–0.97), indicating of an adequate psychometric propertied.

#### Sex invariance measurement

According to the multigroup CFA results (Table 4), we did not verify invariance for sex groups based on the comparisons of CFIs, *χ*^2^, and the degrees of freedom across the unconstrained and constrained models examined.

#### Components correlation

Subsequently, we assessed the correlation between the items of the SMAQ-PT using Spearman rank correlation coefficient. There was a significantly low number of significant correlations (16.5% of all correlations, 20 out of 121; Supplementary Table S3). The correlation between the subscales was also performed, confirming significant correlations between Medical Management with Role Management and Emotional Management, but Role Management did not correlate with Emotional Management (Table 5).

**Table 5:** Correlation between SMAQ-12-PT subscales scores.

SMAQ-12-PT subscale	Medical Management	Role Management	Emotional Management
Medical Management	1.00	0.41⁎⁎⁎	0.23⁎⁎⁎
Role Management	-	1.00	0.11
Emotional Management	-	-	1.00

Values are the Spearman rank correlation coefficient (rho).

⁎⁎⁎ *P* < .001 mean statistical significance.

## Discussion

We have successfully cross-culturally adapted and validated the Portuguese version of the 12-item SMAQ, showing adequate psychometric property on the participants’ view to report ability to self-manage periodontitis. Overall, we found adequate internal consistency and reliability. At the subscale level, reliability and validity remain.

As known, periodontitis is a chronic noncommunicable disease characterised by the accumulation of dental plaque and the subsequent establishment of an altered oral microbiome (oral disbyosis), followed by an exacerbated host-immune response.^31^ Despite its microbiological aetiology, periodontitis relies heavily on the patient behaviours and habits towards oral hygiene, such as poor oral care and smoking, which can significantly worsen their disease and compromise its management.^32^ To effectively address periodontitis through nonsurgical standard therapy, a combination of self-management and professional care is necessary. Self-management entails implementing motivational and behavioural changes to achieve optimal oral hygiene practices (controlling gingival inflammation) and assessing local and systemic modifiable risk factors that may contribute to disease exacerbation (such as active smoking).^33^ Whereas professional care involves removing supra- and, mainly, subgingival plaque and calculus, eliminating local retentive factors is critical for effective and long-term stable disease management.^34^ In fact, self-management of periodontitis and patient cooperation are keystone for the ongoing treatment. Less motivated patients tend to accumulate more interdental plaque and have greater gum bleeding, which explains why professional care alone is not sufficient to control the disease.^35^

The development of a validated Portuguese version of the SMAQ-12-PT has several implications for individual and public health. Self-management ability is an important factor in the controlling of chronic diseases such as periodontitis, which requires active involvement of patients in maintaining good oral hygiene practices and adherence to treatment plans.^36^ The development of a validated Portuguese version of the questionnaire will allow clinicians to better understand the self-management skills of Portuguese-speaking patients with periodontitis, which may lead to more accurate diagnoses, tailored treatment plans, and improved patient outcomes.

Improved patient outcomes can have a significant impact on both individual and public health. Patients with better self-management skills are more likely to adhere to treatment plans, comply with medication regimens, and adopt healthy lifestyle habits, which can ultimately lead to improved oral and systemic health outcomes.^37^ In addition, improved patient outcomes can lead to reduced health care costs, reduced health care utilisation, and improved quality of life.^37^

The development of a validated Portuguese version of the questionnaire may also lead to increased patient satisfaction, which is an important factor in health care delivery. Patients who feel understood and supported by their health care providers are more likely to be satisfied with their care, which can lead to improved patient–provider communication, better patient engagement in their own care, and increased adherence to treatment plans.^38^

Finally, the cross-cultural adaptation and validation of the Portuguese version of the SMAQ-Short Form for periodontitis has important public health implications. By improving the diagnosis and treatment of periodontitis through the use of a validated Portuguese version of the questionnaire, public health officials can better address this important public health issue, leading to improved oral and systemic health outcomes for the Portuguese-speaking population.^39^

### Strengths and limitations

Concerning the strengths of this study, the translation into Portuguese version of SMAQ-12 was carried out by experts following a rigorous linguistic strategy in order to keep the Portuguese version an equivalent measure to the original English questionnaire, without compromising content validity. The short form may contribute to a high level of interest in its application in the daily clinical practice. In addition, we used the most recent periodontitis case definition, which strengthens the validity of clinical screening or public health surveillance in the future. The mode of questionnaire administration was the traditional self-administration method, which reduces the interviewer bias and increases respondents’ willingness to disclose sensitive information.

Despite all strengths, this study had some important limitations worth discussing. First, the sample was a consecutive pool of patients at the University Dental Clinic, although its location may have contributed to limit the generalisability of our results to the entire Portuguese population. Second, the cross-sectional nature of this study analyses a number of variables at a given point in time, but does not provide any information regarding the influence of time on the variables measured. In the periodontitis scenario, time may be crucial to reveal the efficacy of self-management to maintain periodontal health over a lifetime, even with a reduced periodontium.

## Conclusions

The SMAQ-12-PT showed reliability, internal consistency, and construct validity to measure self-periodontitis patient management skills.

## Author contributions

JB analysed and interpreted the data and completed the writing. EG, CI, PL, MM, RCA, JJM, JB, and VM took part in the investigation. EG, CI, JB, and VM made contributions to conceptualisation, participant recruitment, and project management. JB and VM guided the methodology. EG, CI, PL, MM, RCA, JJM, JB, and VM revised the manuscript. JB and VM equally contributed as last authors. All authors have read and approved the final manuscript.

## Funding

This work is financed by national funds through the FCT—Foundation for Science and Technology, I.P., under the Project UIDB/04585/2020.

## Conflict of interest

None disclosed.

## Appendix Supplementary materials

Image, application 1

## References

[1] Albuquerque BN, Cota LOM, Lorentz TCM, Costa FO. (2018). Periodontal maintenance therapy in a public university: a six-year prospective study. J Int Acad Periodontol.

[2] Bäumer A, El Sayed N, Kim TS, Reitmeir P, Eickholz P, Pretzl B (2011). Patient-related risk factors for tooth loss in aggressive periodontitis after active periodontal therapy: patient-related factors for tooth loss in aggressive periodontitis. J Clin Periodontol.

[3] Chambrone L, Chambrone D, Lima LA, Chambrone LA. (2010). Predictors of tooth loss during long-term periodontal maintenance: a systematic review of observational studies: tooth loss during periodontal maintenance. J Clin Periodontol.

[4] Dannewitz B, Zeidler A, Hüsing J (2016). Loss of molars in periodontally treated patients: results 10 years and more after active periodontal therapy. J Clin Periodontol.

[5] Díaz-Faes L, Guerrero A, Magán-Fernández A, Bravo M, Mesa F. (2016). Tooth loss and alveolar bone crest loss during supportive periodontal therapy in patients with generalized aggressive periodontitis: retrospective study with follow-up of 8 to 15 years. J Clin Periodontol.

[6] Goh V, Hackmack P, Corbet E, Leung W. (2017). Moderate- to long-term periodontal outcomes of subjects failing to complete a course of periodontal therapy. Aust Dent J.

[7] Pretzl B, El Sayed S, Weber D, Eickholz P, Bäumer A (2018). Tooth loss in periodontally compromised patients: results 20 years after active periodontal therapy. J Clin Periodontol.

[8] Eickholz P, Kaltschmitt J, Berbig J, Reitmeir P, Pretzl B. (2008). Tooth loss after active periodontal therapy. 1: patient-related factors for risk, prognosis, and quality of outcome: patient-related factors for tooth loss. J Clin Periodontol.

[9] Kim SY, Lee JK, Chang BS, Um HS. (2014). Effect of supportive periodontal therapy on the prevention of tooth loss in Korean adults. J Periodontal Implant Sci.

[10] Needleman I, Garcia R, Gkranias N (2018). Mean annual attachment, bone level, and tooth loss: a systematic review. J Clin Periodontol.

[11] Carvalho R, Botelho J, Machado V (2021). Predictors of tooth loss during long-term periodontal maintenance: an updated systematic review. J Clin Periodontol.

[12] Corbin JM, Strauss AL. (1988).

[13] Li M, He S, Wang J. (2022). Development and validation of a new short form of the self-management ability questionnaire for patients with chronic periodontitis. Comm Dent Oral Epid.

[14] Lhakhang P, Hamilton K, Sud N (2016). Combining self-management cues with incentives to promote interdental cleaning among Indian periodontal disease outpatients. BMC Oral Health.

[15] Chapple ILC, Van der Weijden F, Doerfer C (2015). Primary prevention of periodontitis: managing gingivitis. J Clin Periodontol.

[16] Chen C, Feng X, Li YT, Zhang Q, Jin YS. (2019). Development and validation of a self-management ability questionnaire for patients with chronic periodontitis. Int J Nurs Sci.

[17] Lindhe J, Nyman S. (1977). The role of occlusion in periodontal disease and the biological rationale for splinting in treatment of periodontitis. Oral Sci Rev.

[18] Hamp SE, Nyman S, Lindhe J. (1975). Periodontal treatment of multirooted teeth. Results after 5 years. J Clin Periodontol.

[19] Tonetti MS, Greenwell H, Kornman KS. (2018). Staging and grading of periodontitis: framework and proposal of a new classification and case definition. J Periodontol.

[20] Terwee CB, Bot SDM, de Boer MR (2007). Quality criteria were proposed for measurement properties of health status questionnaires. J Clin Epidemiol.

[21] DeVon HA, Block ME, Moyle-Wright P (2007). A psychometric toolbox for testing validity and reliability. J Nursing Scholarship.

[22] Weir JP. (2005). Quantifying test-retest reliability using the intraclass correlation coefficient and the SEM. J Strength Cond Res.

[23] Tabachnick BG, Fidell LS. (2007).

[24] Steiger JH. (2007). Understanding the limitations of global fit assessment in structural equation modeling. Pers Individ Differ.

[25] Byrne BM. (2013). https://www.taylorfrancis.com/books/9780203807644.

[26] Shevlin M, Miles JNV. (1998). Effects of sample size, model specification and factor loadings on the GFI in confirmatory factor analysis. Pers Individ Differ.

[27] Hu L, Bentler PM. (1999). Cutoff criteria for fit indexes in covariance structure analysis: conventional criteria versus new alternatives. Struct Equ Modeling.

[28] Dimitrov DM. (2010). Testing for factorial invariance in the context of construct validation. Meas Eval Couns Dev.

[29] Bollen KA. (1989,).

[30] Satorra A, Bentler PM. (2010). Ensuring positiveness of the scaled difference Chi-square test statistic. Psychometrika.

[31] Könönen E, Gursoy M, Gursoy U. (2019). Periodontitis: a multifaceted disease of tooth-supporting tissues. J Clin Med.

[32] Kassebaum NJ, Bernabé E, Dahiya M, Bhandari B, Murray CJL, Marcenes W. (2014). Global burden of severe periodontitis in 1990-2010: a systematic review and meta-regression. J Dent Res.

[33] Suvan J, Leira Y, Moreno Sancho FM, Graziani F, Derks J, Tomasi C (2020). Subgingival instrumentation for treatment of periodontitis. A systematic review. J Clin Periodontol.

[34] Sanz M, Herrera D, Kebschull M (2020). Treatment of stage I–III periodontitis—the EFP S3 level clinical practice guideline. J Clin Periodontol.

[35] Oruba Z, Pac A, Olszewska I, Chomyszyn-Gajewska M. (2014). The significance of motivation in periodontal treatment: the influence of adult patients’ motivation on the clinical periodontal status. Community Dent Health.

[36] Barker I, Steventon A, Williamson R, Deeny SR. (2018). Self-management capability in patients with long-term conditions is associated with reduced healthcare utilisation across a whole health economy: cross-sectional analysis of electronic health records. BMJ Qual Saf.

[37] Bodenheimer T, Lorig K, Holman H, Grumbach K. (2002). Patient self-management of chronic disease in primary care. JAMA.

[38] Kennedy BM, Rehman M, Johnson WD, Magee MB, Leonard R, Katzmarzyk PT (2017). Healthcare providers versus patients’ understanding of health beliefs and values. Patient Exp J.

[39] Machado V, Lyra P, Santos C, Proença L, Mendes JJ, Botelho J. (2022). Self-reported measures of periodontitis in a Portuguese population: a validation study. J Pers Med.

