# Supplementary material for: Self-Management Ability Questionnaire Validation in Portuguese Adults With Periodontitis
Source: Int Dent J. 2023 Jul 11;73(6):889–95. doi: 10.1016/j.identj.2023.06.003 (PMC10658420; doi:10.1016/j.identj.2023.06.003)
Supplement: Supplementary file 1 [file mmc1.docx]

Supplementary Material

## Supplementary Figures

## Supplementary Table 1. Original and Portuguese versions of the SMAQ-12 questionnaire.

| **Item** | **Original** | **Portuguese** |
| --- | --- | --- |
| 1 | I use interdental brush/dental floss to clean my teeth when I suffer from chronic periodontitis. | Uso escovilhão interdentário/fio dentário para higienizar os meus dentes quando sofro da periodontite. |
| 2 | I brush my teeth/rinse my mouth after every meal. | Escovo os meus dentes/bochecho a minha boca após cada refeição. |
| 3 | I brush the space between the gum and the teeth. | Escovo o espaço entre a gengiva e os dentes. |
| 4 | I go to the dentist following a dentist’s advice, when I suffer from chronic periodontitis. | Vou ao dentista seguindo o conselho de um dentista, quando eu sofro da periodontite. |
| 5 | I obtain information about chronic periodontitis through internet/books/lectures. | Obtenho informações sobre periodontite através da internet/livros/palestras. |
| 6 | I take calcium- and vitamin D-rich foods, such as dried small shrimp, fish, and milk | Como alimentos ricos em cálcio e vitamina D, como como peixe e leite. |
| 7 | I deal with food impaction just in time. | Lido de imediato com a retenção de alimentos entre os dentes. |
| 8 | I do not eat food that can damage my teeth, such as betel nut, carbonated drink and coffee | Não como alimentos que possam danificar os meus dentes, como nozes, refrigerante e café. |
| 9 | I avoid habits that can damage teeth, such as sucking fingers, clenching teeth, biting nails, and biting pencils. | Evito hábitos que possam danificar os dentes, como chuchar no dedo, cerrar os dentes, roer unhas e morder o lápis/caneta. |
| 10 | I share how I feel with others when my periodontal tissue is unwell and triggers a negative emotion. | Quando as minhas gengivas não estão bem e isso tem um impacto negativo em mim, eu partilho com outras pessoas. |
| 11 | I divert my attention when my periodontal tissue is unwell and triggers a negative emotion. | Quando as minhas gengivas não estão bem e isso tem um impacto negativo em mim, tento ignorar. |
| 12 | I stay optimistic even though I suffer from chronic periodontitis. | Continuo otimista mesmo sofrendo da periodontite. |

These twelve items are rated on a response scale: 1 = “Never”, 2 = “Rarely”, 3 = “Sometimes”, 4 = “Often”, and 5 = “Very often” (in Portuguese 1= “Nunca”; 2 =”Raramente”; 3 = “Às vezes”; 4 = “Frequente”; 5 = “Muito frequente”).

## Supplementary Table 2. Test–retest reliability using ICCs for the SMAQ-PT questionnaire.

|  | **Cronbach’s α coefficient**  **(95% CI)** | **ICC (95% CI)** | **p-value** |
| --- | --- | --- | --- |
| Item 1 | 0.93 (0.84; 0.97) | 0.87 (0.73; 0.94) | <0.001 |
| Item 2 | 0.81 (0.62; 0.92) | 0.68 (0.42; 0.84) | <0.001 |
| Item 3 | 0.86 (0.54; 0.96) | 0.77 (0.55; 0.89) | <0.001 |
| Item 4 | 0.93 (0.77; 0.99) | 0.87 (0.74; 0.94) | <0.001 |
| Item 5 | 0.88 (0.73; 0.96) | 0.76 (0.50; 0.89) | <0.001 |
| Item 6 | 0.89 (0.75; 0.96) | 0.79 (0.60; 0.90) | <0.001 |
| Item 7 | 0.96 (0.89; 1.00) | 0.93 (0.86; 0.97) | <0.001 |
| Item 8 | 0.86 (0.66; 0.94) | 0.76 (0.55; 0.89) | <0.001 |
| Item 9 | 0.87 (0.10; 0.97) | 0.59 (0.28; 0.79) | <0.001 |
| Item 10 | 0.91 (0.70; 0.95) | 0.78 (0.57; 0.89) | <0.001 |
| Item 11 | 0.85 (0.54; 0.97) | 0.74 (0.59; 0.85) | <0.001 |
| Item 12 | 0.79 (0.46; 0.96) | 0.66 (0.38; 0.83) | <0.001 |
| SMAQ-12 | 0.90 (0.77; 0.97) | 0.90 (0.79; 0.95) | <0.001 |

CI - confidence interval; ICC - intraclass correlation coefficient.

## Supplementary Table 3. Correlation between SMAQ-12 item scores.

| **Item** | **1** | **2** | **3** | **4** | **5** | **6** | **7** | **8** | **9** | **10** | **11** | **12** |
| --- | --- | --- | --- | --- | --- | --- | --- | --- | --- | --- | --- | --- |
| **1** | 1.000 | 0,74 ** | 0,96 *** | 0,82 *** | 0,36 | 0,63 * | 0,57 | 0,2 | 0,56 | 0,34 | -0,1 | 0,56 |
| **2** | - | 1.000 | 0,88 *** | 0,83 *** | -0,08 | 0,73 ** | 0,83 *** | 0,33 | 0,02 | 0,08 | -0,38 | 0,62 * |
| **3** | - | - | 1.000 | 0,85 *** | 0,15 | 0,74 ** | 0,71 * | 0,18 | 0,36 | 0,18 | -0,2 | 0,64 * |
| **4** | - | - | - | 1.000 | 0,1 | 0,67 * | 0,58 * | 0,1 | 0,24 | 0,29 | -0,38 | 0,83 *** |
| **5** | - | - | - | - | 1.000 | -0,06 | 0,03 | 0,38 | 0,79 *** | 0,92 *** | -0,08 | -0,26 |
| **6** | - | - | - | - | - | 1.000 | 0,86 *** | -0,02 | 0,06 | 0,03 | -0,5 | 0,73 ** |
| **7** | - | - | - | - | - | - | 1.000 | 0,23 | -0,02 | 0,17 | -0,49 | 0,47 |
| **8** | - | - | - | - | - | - | - | 1.000 | 0,29 | 0,42 | -0,24 | -0,29 |
| **9** | - | - | - | - | - | - | - | - | 1.000 | 0,66 * | 0,13 | 0,01 |
| **10** | - | - | - | - | - | - | - | - | - | 1.000 | -0,3 | -0,08 |
| **11** | - | - | - | - | - | - | - | - | - | - | 1.000 | -0,24 |
| **12** | - | - | - | - | - | - | - | - | - | - | - | 1.000 |

* *p* < 0.05, ** *p* < 0.01, *** *p* < 0.001.
